# Thermal decomposition behavior and kinetics for pyrolysis and catalytic pyrolysis of Douglas fir

**DOI:** 10.1039/c7ra12187c

**Published:** 2018-01-09

**Authors:** Lu Wang, Hanwu Lei, Jian Liu, Quan Bu

**Affiliations:** School of Biological and Medical Engineering, Hefei University of Technology Hefei 230009 China wanglu@hfut.edu.cn +86-551-62901331; Bioproducts, Sciences and Engineering Laboratory, Department of Biological Systems Engineering, Washington State University Richland WA 99354-1671 USA hlei@tricity.wsu.edu +1-509-372-7690 +1-509-372-7628; Institute of Agricultural Engineering, Jiangsu University Zhenjiang 212013 China

## Abstract

In this study, the thermal decomposition behavior and kinetics of pyrolysis and catalytic pyrolysis of Douglas fir (DF) were investigated using thermogravimetric (TG) analysis. It was found that the heating rate was an important factor during the biomass pyrolysis process, it affected the pyrolysis though heat transfer and mass transfer through the biomass particles. The differential thermogravimetric (DTG) curves demonstrated that the role of the catalyst was to slightly reduce the temperature of biomass thermal degradation. We obtained the thermal data including the activation energy, frequency factor and reaction order by Coats–Redfern and Friedman methods. For the Coats–Redfern method, we found that the activation energy of the catalytic pyrolysis was lower than that of the non-catalytic pyrolysis. It means that the ZSM-5 catalyst increased the rate of reaction and reduced the energy required for the decomposition process. Meanwhile, the result from the Friedman method demonstrated that the reaction could be divided into two steps, which were reaction rate between 0.2 and 0.7 and a reaction rate of 0.8 based on parallelism. Addition of the ZSM-5 catalyst reduced the activation energy in the first region then increased it in the second region due to the secondary cracking of intermediate compounds which was highly affected by shape-selective catalysis. Simulation of pyrolysis and catalytic pyrolysis of DF using the obtained kinetic parameters was in good agreement with the experimental data. Py-GC/MS analysis was also carried out and indicated that the ZSM-5 catalyst had a highly positive effect on aromatic hydrocarbon production by significantly reducing oxygen-containing compounds (*i.e.* acids, esters, ketones/aldehydes and guaiacols) during the catalytic pyrolysis of DF.

## Abstract 2

Investigation of non-catalytic and catalytic pyrolysis of DF by thermogravimetric analysis revealed that the application of ZSM-5 reduced the activation energy.

## Graphical abstract

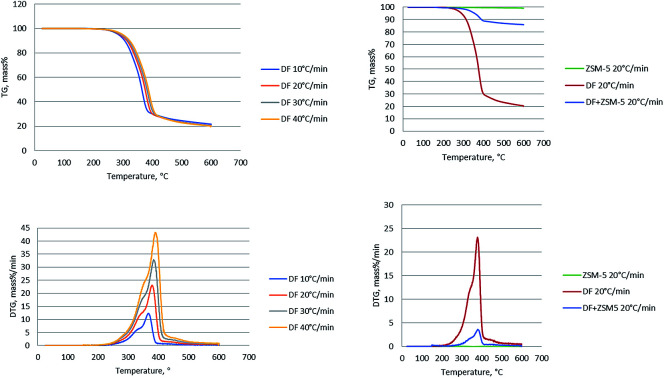

## 1. Introduction

Due to its abundance and low cost, biomass has been realized as one of the most significant sustainable alternatives to petroleum fuels. Pyrolysis conversion is one of the most promising methods that can directly convert biomass into liquid fuels called bio-oils, which runs at 350–600 °C in the absence of oxygen.^1^ It draws much attention because of its short residence time,^2^ easy separation of products,^2^ and wide range of feedstock^3,4^ compared to other methods. Bio-oil is a complex mixture of sugars, esters, furans, acids, ketones, alcohols, phenols, guaiacols, and so on.^5^ It has high oxygen content and is acidic, viscous, reactive, and thermally unstable, and thus cannot be directly used in engines or traditional refineries. It has been found that the oxygen content of bio-oils is usually 35–40%, which is one of the most significant differences between bio-oils and petroleum fuels.^6,7^ The high oxygen content therefore makes the bio-oils incompatible with the existing petroleum-derived oils.^8^

In order to use bio-oil for transport fuel application, upgrading technologies by using cracking catalyst on pyrolysis vapors are of interest to reduce the oxygen contents *via* dehydration and decarboxylation reactions and also obtain higher hydrocarbon in bio-oil.^9^ Catalytic fast pyrolysis is a modified fast pyrolysis method to produce aromatic hydrocarbons such as benzene, toluene, and xylenes by pyrolyzing biomass in the presence of catalyst.^10,11^ The influence of the catalyst was to convert the oxygen in the pyrolysis oil to H_2_O, CO and CO_2_. For example, the bio-oil yield was markedly reduced in the presence of ZSM-5 zeolite catalyst, while the oxygen content of the bio-oil reduced when the rice husks were pyrolyzed with the catalyst.^12^ This process has several advantages over the traditional method such as using a single reactor, inexpensive catalysts and gasoline miscible products.

Understanding the kinetics of pyrolysis and catalytic pyrolysis is vital to design, optimize, and scale up industrial biomass conversion applications. TGA is the most commonly applied thermo analytical technique for thermal study of biomass pyrolysis.^12^ TGA measures the decrease in substrate mass caused by the release of volatiles during thermal decomposition as a function of time^13^ and the pyrolysis kinetics of many kinds of biomass^12,14–16^ have been studied using TGA method. There are also several studies have been done on kinetics of catalytic pyrolysis such as tobacco rob mixed with catalyst (dolomite and NiO),^17^ corn stalk with sodium carbonate or potassium carbonate as catalyst,^18^ wheat straws with three kinds of catalyst (*i.e.* solid acid catalyst, bifunctional catalyst, and industrial catalyst).^19^

Douglas fir (DF) used as feedstock in our previous work is one of the most widespread and abundant species in western North America, which contains 44% cellulose, 21% hemicelluloses and 32% lignin^20^ and is regarded as an important biomass resource. However, very few works has been reported on the kinetics of catalytic pyrolysis of DF. Therefore, the aim of this study was to investigate both the pyrolysis and catalytic pyrolysis behavior of DF through TGA and to develop their kinetic models using Coats–Redfern and Friedman methods.

## 2. Materials and methods

### 2.1 Materials

The feedstock used in this study was DF pellet (Bear Mountain Forest Products Inc., USA), which were approximately 5 mm in diameter and 20 mm in length with moisture content of 8%. The DF pellet was grinded into small particle size (1–2 mm) before using. Catalyst ZSM-5 (Zeolyst International, USA; SiO_2_/Al_2_O_3_ mole ratio: 50) was dried at 105 °C for 12 h and calcined in a muffle furnace at 550 °C for 5 h. The treated catalyst was pelletized and sieved to 100 mesh.

### 2.2 Thermogravimetric analysis (TGA)

The thermal degrading behavior of DF pyrolysis was analyzed by a TG analyzer (Mettler Toledo 188 TGA/SDTA 851, Switzerland). For each test, about 8 mg sample was loaded into an alumina crucible and heated from 25 to 600 °C at selected heating rates (10, 20, 30, and 40 °C min^−1^) with a nitrogen flow rate of 20 mL min^−1^.

The same TG analyzer was used to perform the TGA for catalysis pyrolysis of DF with ZSM-5 as catalyst. For each test, about 8 mg (DF and ZSM-5 was blended with the mass ratio of 1 : 3) sample was loaded into an alumina crucible and heated from 25 to 600 °C at selected heating rates (10, 20, 30, and 40 °C min^−1^) with a nitrogen flow rate of 20 mL min^−1^.

### 2.3 Kinetic study

Under isothermal conditions, the reaction rate is commonly described by the following equation as:

1 d*α*/d*t* = *kf*(*α*)

where *k* is reaction rate, *t* is reaction time (s), *n* is reaction order, and *α* is defined in terms of the change in mass of samples:

2 *α* = (*x*_0_ − *x*)/(*x*_0_ − *x*_f_)

The reaction rate of decomposition is a function of temperature, usually given by the Arrhenius equation:

3 *k* = *A* exp(−*E*/*RT*)

where *A* is the pre-exponential factor (s^−1^), *E* is the activation energy (J mol^−1^) defined as the energy barrier before molecules can get close enough to react and form products.^21^*R* is universal gas constant (8.3145 J mol^−1^ K^−1^), and *T* is the temperature (K).

Substituting the reaction rate (eqn (3)) into eqn (1) gives the following equation:

4 d*α*/d*t* = *A* exp(−*E*/*RT*)*f*(*α*)

For a constant heating rate, *β* (K s^−1^) may be defined as:

5 *β* = d*T*/d*t*

Finally, inserting heating rate (eqn (5)) into eqn (4) gives the final equation:

6 d*α*/d*t* = *β*(d*α*/d*T*) = *A* exp(−*E*/*RT*)*f*(*α*)

Since numbers of simultaneous reactions were involved in the biomass decomposition process, in order to solve eqn (6) for the activation energy (*E*) and the frequency factor (log *A*), two methods were adopted in this study, one is a model-free method and the other one is a model-fitting method.

#### 2.3.1 Model-fitting method

The integral method of Coats–Redfern^22^ derived from the Arrhenius equation has been widely applied for kinetics analysis of solid decomposition. Separating variables in eqn (6) gives:

7 d*α*/*f*(*α*) = d*α*/(1 − *α*)^*n*^ = (*A*/*β*) × exp(−*E*/*RT*)d*T*

Integrating between the limits: *α* = 0 at *T* = *T*_0_ and *α* = *α* at *T* = *T*_*α*_ gives:8
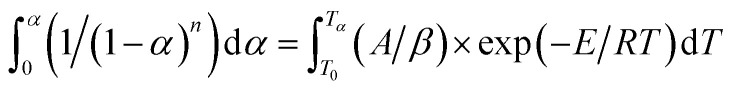


Integrating (1 − *α*)^*n*^ and exp(−*E*/*RT*) in eqn (8), the following expression can be obtained:9
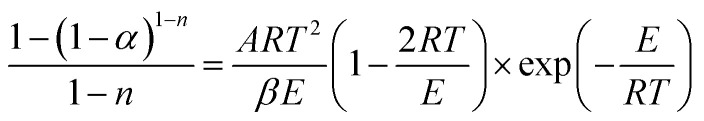


Transforming eqn (9) into a logarithmic expression:10
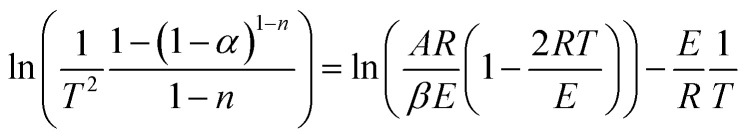


Assuming (1 − 2*RT*/*E*) ≈ 1, eqn (10) becomes:11
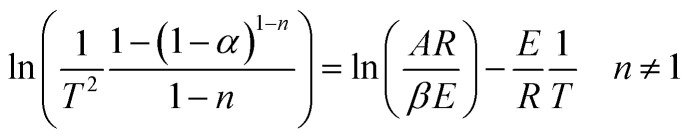
12
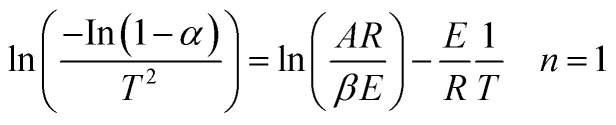


Therefore, a straight line with slope = *E*/*R* and intercept = ln(*AR*/*βE*) can be figured out.

#### 2.3.2 Model-free method

Model-free method was proposed on an isoconversional basis where the degree of the conversion was assumed to be constant and the reaction rate was dependent on the reaction temperature.^23^ There are several model-free methods, such as Friedman,^24^ FWO,^25^ and KAS.^26^ Among them, Friedman method, as one of the first proposed isoconversional methods, could be expressed by converting eqn (6) into a logarithmic expression:

13 ln(d*α*/d*t*) = ln[(*Af*(*α*))] − *E*/*RT*

According to Friedman's kinetic method, conversion function is assumed to be constant, which means that it only depends on the mass loss rate. Therefore, for the given value of conversion rate, the plot ln(d*α*/d*t*) *versus* 1/*T* gives a straight line with the slope of −*E*/*R*.

### 2.4 Pyrolysis-GC/MS

Py-GC/MS analyses were carried out on a CDS pyroprobe 5000 series (CDS Analytical, Inc.), which is connected to a GC/MS system (6890N Network GC System, 5975B inert XL MSD, Agilent Technologies). Approximately 500 μg of the feedstock (DF and DF + ZSM-5) were used. As for the catalytic pyrolysis, DF and ZSM-5 was mixed with the mass ratio of 1 : 3. The oven temperature was set to 270 °C and the filament was heated to 500 °C. The GC inlet temperature was set up at 250 °C. The GC oven temperature was set at 40 °C, held for 1 min and then heated at a rate of 6 °C min^−1^ to 280 °C. The oven was held at the final temperature for 15 min. Helium was used as carrier gas at a flow rate of 1 mL min^−1^ in split mode with split ratio of 50 : 1.

## 3. Results and discussion

### 3.1 Pyrolysis and catalytic pyrolysis behavior of DF

**Fig. 1:**
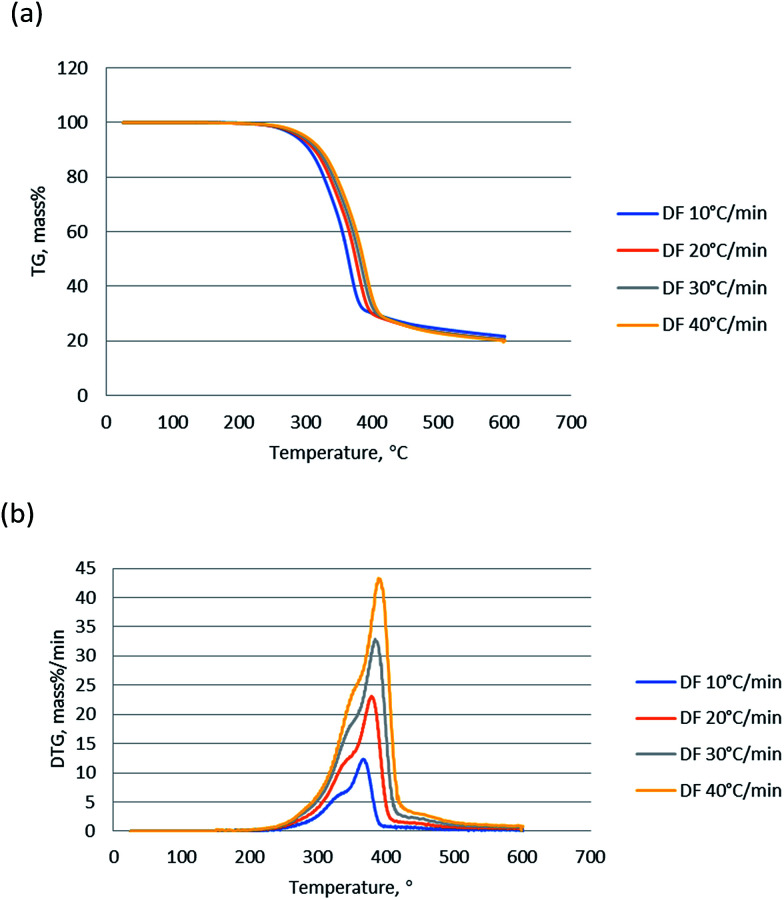
(a) TG and (b) DTG curves of non-catalytic pyrolysis of DF at different heating rates.

Thermogravimetric analysis showed the relationship between the weight change of a sample and temperature, playing a significant role in understanding of the thermal decomposition and reaction mechanism during the pyrolysis. TG curves indicates the mass loss of the sample *versus* temperature change of the thermal degradation, and DTG presents the corresponding rate of mass loss of TG curves. Fig. 1 shows the TG and DTG curves of DF pyrolysis at four different heating rates (10, 20, 30, and 40 °C min^−1^). The changes (5% mass%) at the temperature lower than 150 °C was not counted since it was attributed to vaporization of moisture.^27^ TG curves show that the main weight loss of DF was between 250 and 430 °C, which was a result of the primary decomposition reaction.^28^ Meanwhile, there are two peaks around 340 °C and 380 °C and a long tail at high temperature in the DTG curves. The 340 °C peak was mainly contributed by hemicellulose devolatilization, and the second 380 °C was mainly attributed to cellulose devolatilization.^29,30^ The long tail at high temperatures was corresponding to the thermal decomposition of lignin, due to the very board decomposition range.^31^

The heating rate is an important factor during biomass pyrolysis process, since it affects the pyrolysis though heat transfer and mass transfer through the biomass particles. As shown in Fig. 1, the shape of TG and DTG curves at various rates is almost the same and there is no significant difference in pyrolysis residues. Nevertheless, the peaks of DTG curve became sharper as the heating rate increased, and the weight loss rates were significantly enhanced by higher heating rates compared to those by the lower heating rates. And the recoded peak weight loss rates were 12.2, 23.1, 32.9 and 42.5 mass% per min, for 10, 20, 30 and 40 °C min^−1^ respectively. The Fig. 1 shows that the peaks of DTG curves slightly shifted to the right as the heating rate increased, due to the fact that gradient of temperature of a particle and distribution of temperature was smaller at low heating rates.

**Fig. 2:**
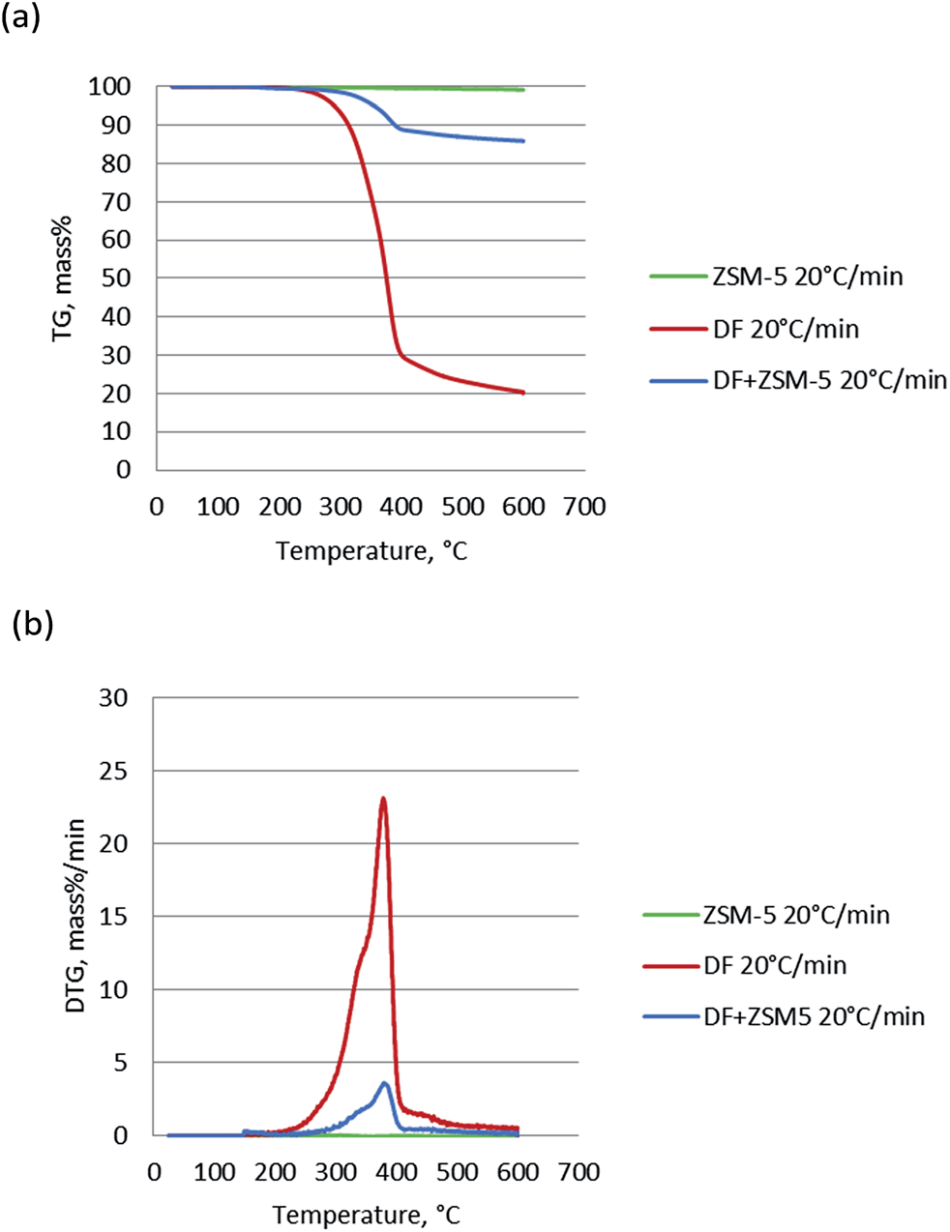
(a) TG and (b) DTG curves of non-catalytic pyrolysis and catalytic pyrolysis of DF.

The TG and DTG of pure ZSM-5 catalyst, DF, and DF + ZSM-5 (DF and ZSM-5 blended with the mass ratio of 1 : 3) were performed and the results are shown in Fig. 2. For the pure ZSM-5 catalyst, the 1.4% weight loss below 150 °C due to vaporization of moisture was not counted and hence the total weight loss from 150 °C to 600 °C was only 0.8%, negligible when compared with the weight loss of DF (∼80%). Comparing the TG and DTG curves of the DF and DF + ZSM-5, it can be seen that in the presence of the catalyst, the peaks of DTG curves slightly shifted to the left. This means the addition of the catalyst tends to slightly lower the temperature of thermal degrading process, in agreement with the results reported by Yang^17^ from the pyrolysis of tobacco rob using dolomite and NiO as catalyst.

### 3.2 Decomposition kinetics

#### 3.2.1 Model-fitting method

The activation energy (*E*) was calculated from the heating rate at 20 °C min^−1^ and listed in Table 1. The reaction orders were varied and kinetic parameters were recalculated for each order. As shown in the Table 1, when the reaction order increased, the calculated activation energy also increased. The highest regression coefficients (*R*^2^ = 0.976) were achieved for the DF pyrolysis with the activation energy as 78.15 kJ mol^−1^. And for the DF catalytic pyrolysis, the activation energy was 65.11 kJ mol^−1^ with the highest *R*^2^ at 0.986. Activation energy is defined as the minimum energy requirement that must be overcome before molecules can get close enough to react and form products.^21^ The activation energy of the catalytic pyrolysis was lower than that of the non-catalytic pyrolysis. It means that the ZSM-5 catalyst increased the rate of reaction, reduced the energy required for decomposition process.

**Table 1:** Activation energy, log *A* and *R*^2^ for DF pyrolysis by Coats–Redfern method

Feedstock	Reaction order	Activation energy (kJ mol^−1^)	*R* ^2^
DF	*n* = 1	78.153	0.976
	*n* = 2	116.72	0.973
	*n* = 3	164.72	0.945
	*n* = 4	218.75	0.917
	*n* = 5	276.41	0.898
	*n* = 6	336.30	0.885
DF + ZSM-5	*n* = 1	65.113	0.986
	*n* = 2	75.723	0.964
	*n* = 3	111.19	0.957
	*n* = 4	159.44	0.950
	*n* = 5	193.63	0.941
	*n* = 6	237.66	0.934

#### 3.2.2 Model-free method

**Fig. 3:**
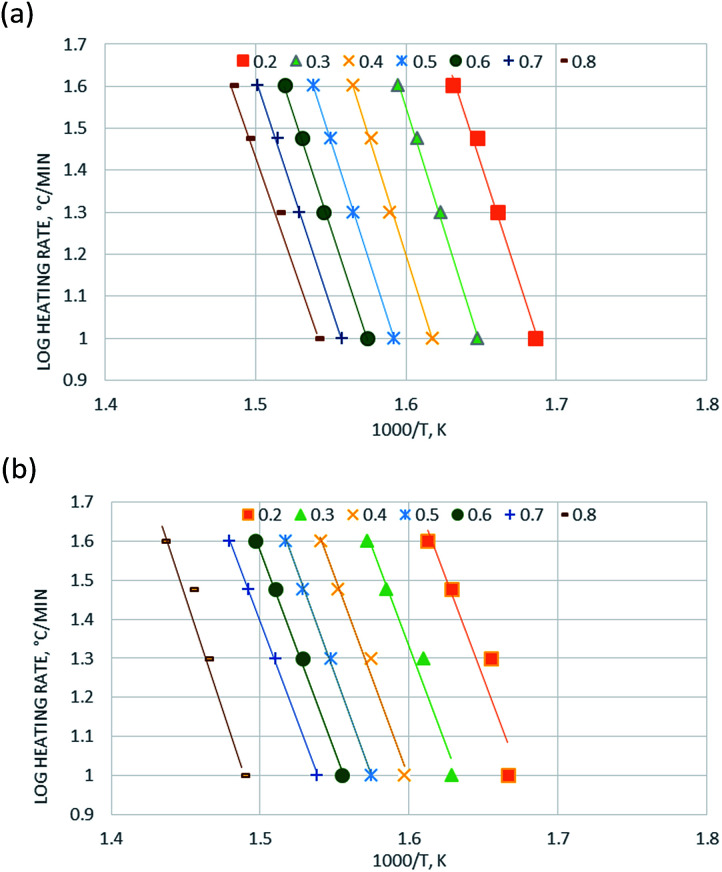
Kinetics analysis of (a) non-catalytic and (b) catalytic pyrolysis of DF by the Friedman method.

There have been ongoing debates about the use of isoconversional methods for the solid state decomposition since the activation energy.^23^ Some researchers^32^ claimed that the model-fitting method might not describe the complex processes such as biomass decomposition sufficiently, since numbers of reactions are involved simultaneously in the process. At this point, model-free approaches play an important role for the investigation of the change in activation energy according to varying conversion. According to Friedman's kinetic method, the activation energies for each conversion rate from 0.2 to 0.8 were plotted in Fig. 3. And the calculated activation energies together with regression coefficients for each conversion rate were calculated and summarized in Table 2. It is clear that each line had good correlation with the selected models since high *R*^2^ values were achieved. And the parallelism of lines was attributed to the similar kinetic behavior probably the same reaction mechanism was achieved.^33^ The average activation energy of catalytic pyrolysis of DF was 192.7 kJ mol^−1^, less than that (204.7 kJ mol^−1^) of non-catalytic pyrolysis, indicating that the average activation energy was reduced in the presence of the ZSM-5 catalyst. The trend of these lines could be divided into two groups: *α* = 0.2–0.7 and *α* = 0.8 based on their parallelism. For the interval of *α* = 0.2–0.7, the apparent activation energies were close to each other and calculated to be 200.55–213.23 kJ mol^−1^ and 186.33–192.06 kJ mol^−1^ for pyrolysis and catalytic pyrolysis respectively. For the next reaction progress (*α* = 0.8), the apparent activation energy was found to be 191.03 kJ mol^−1^ and 214.33 kJ mol^−1^ for pyrolysis and catalytic pyrolysis respectively. The dependence of the activation energies on conversion means that the pyrolysis was a complex process consisting of different reactions.

**Table 2:** Activation energy and *R*^2^ for DF pyrolysis by Friedman method

*α*	DF		DF + ZSM-5	
	*E* (kJ mol^−1^)	*R* ^2^	*E* (kJ mol^−1^)	*R* ^2^
0.2	206.16	0.992	186.33	0.908
0.3	213.25	0.997	186.67	0.964
0.4	211.60	0.997	191.46	0.988
0.5	207.60	0.999	192.06	0.998
0.6	202.74	0.999	190.79	0.997
0.7	200.55	0.998	187.75	0.999
0.8	191.03	0.996	214.33	0.976

**Fig. 4:**
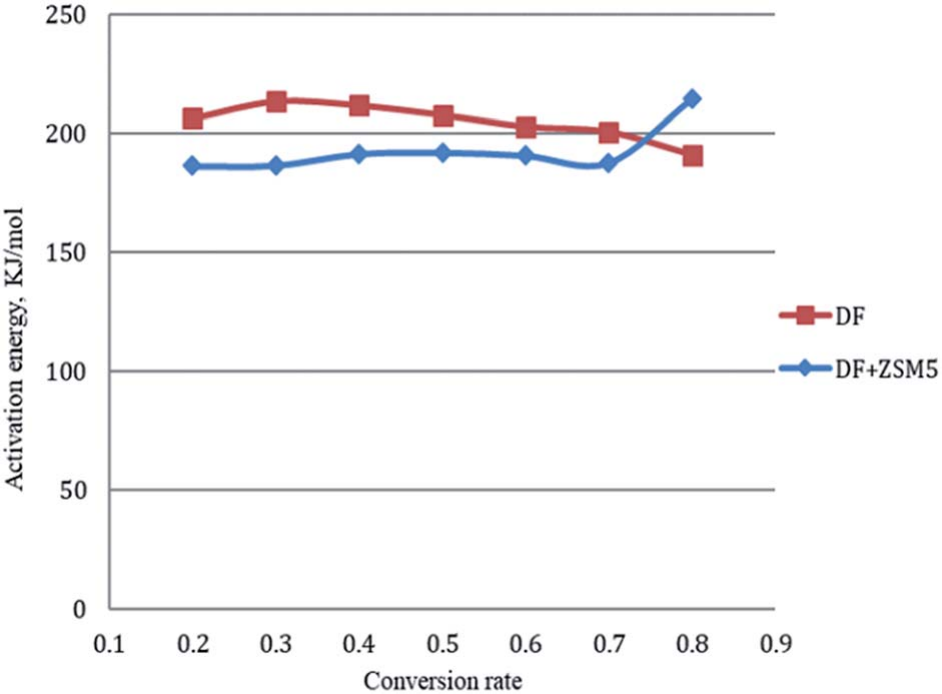
Activation energies for each conversion rate from 0.2 to 0.8 by Friedman method.

The activation energy for each conversion rate for non-catalytic pyrolysis and catalytic pyrolysis was showed in Fig. 4. The apparent activation energy of pyrolysis decreased in the second reaction progress (*α* = 0.8), indicating that the energy required for the final stages of the non-catalytic pyrolysis was lower than that required for the first part. This behavior was in consistent with the results reported in the previous study.^23^ In contrast with the non-catalytic pyrolysis process, the apparent activation energy of the catalytic pyrolysis increased significantly to 214.33 kJ mol^−1^ in the last reaction progress. The reduction of the activation energy in the first region and then the increase of it in the second region could be explained by that at the last stage of the catalytic pyrolysis, the reaction probably included secondary cracking of intermediate compounds, producing small molecules^34^ that were highly affected by shape-selective catalyst used in this study (ZSM-5) and in turn resulted in higher activation energy in the final stage than that in the first stage.

#### 3.2.3 Comparison of two methods

The activation energy calculated from Coats–Redfern were 78.15 kJ mol^−1^ and 65.11 kJ mol^−1^ for DF pyrolysis and catalytic pyrolysis respectively. While using Friedman method, the activation energies were calculated to be 191.03–213.23 kJ mol^−1^ and 186.33–214.33 kJ mol^−1^ for pyrolysis and catalytic pyrolysis respectively. There is difference between the activation energy calculated from two methods. For the Coats–Redfern method, the reaction orders were varied and kinetic parameters were recalculated for each order, resulted in the calculated activation energy increased with the increased reaction order. There have been ongoing debates about the use of isoconversional methods for the solid state decomposition since the activation energy. Some researchers claimed that the model-fitting method might not describe the complex processes such as biomass decomposition sufficiently, since numbers of reactions are involved simultaneously in the process. At this point, model-free approaches played an important role for the investigation of the change in activation energy according to varying conversion. It has been reported^23^ that the activation energy produced by the Friedman method was very close to that by the FWO and KAS methods.

### 3.3 Validation

**Fig. 5:**
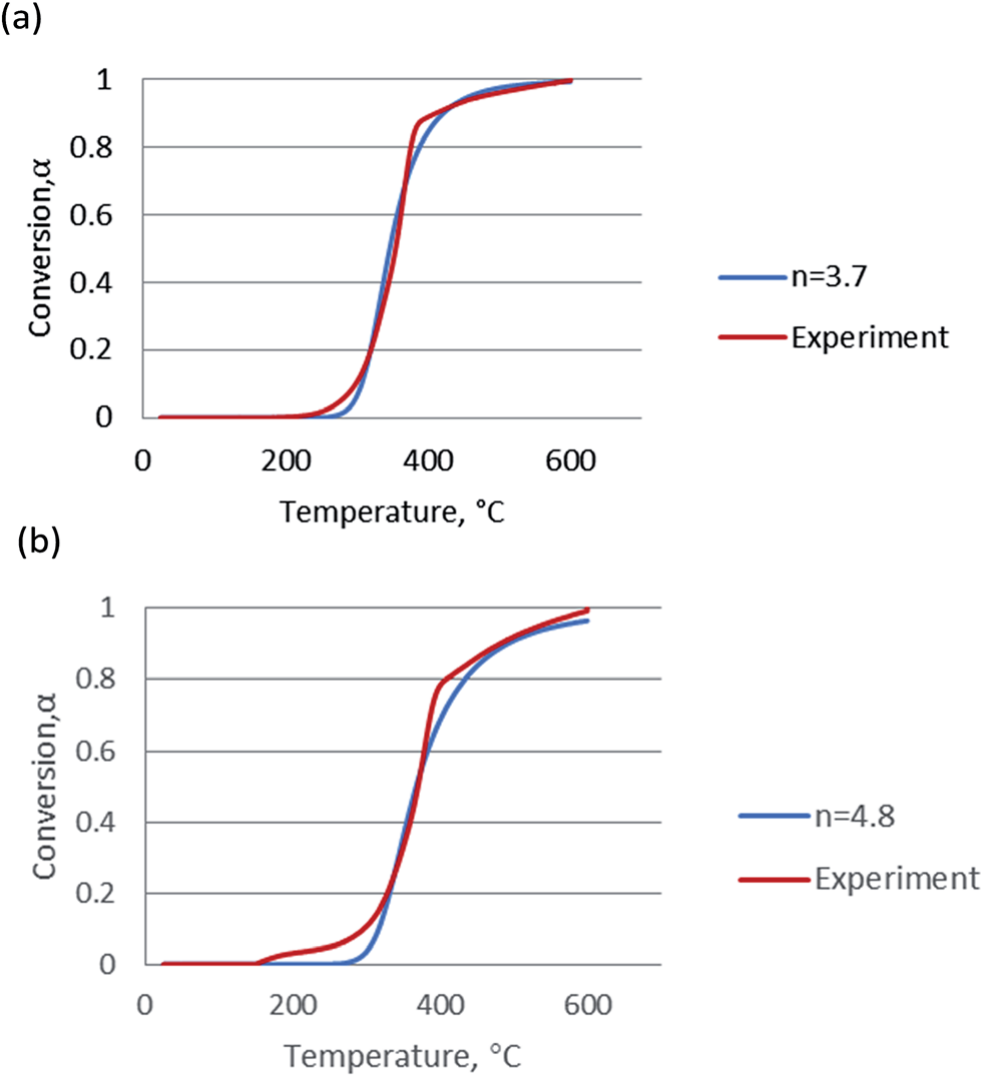
Simulation of (a) non-catalytic and (b) catalytic pyrolysis using the kinetic data calculated from the Friedman method.

Since the activation energy calculated by the Friedman method was very close to that by the FWO and KAS methods and considered to be more accurate, the mean value of activation energies obtained from the Friedman method was used for in Coats–Redfern equations for calculation of frequency factor (log *A*). The plots for *n* = 1, Coats–Redfern model was lack of accuracy for representing experimental results, for *n* ≠ 1, as claimed in these two literatures,^35,36^ the pseudo-order *n* had no physical meaning but played an important role as a correlation parameter to fit parameter of pyrolysis model. In order to validate kinetic parameters, the simulation was carried out for the heating rate at 20 °C min^−1^ and the results were presented in Fig. 5. The calculated data agree with the experimental data very well when *n* = 3.7 for non-catalytic pyrolysis and *n* = 4.8 for catalytic pyrolysis of DF.

### 3.4 Py-GC/MS analysis and mechanism analysis

**Fig. 6:**
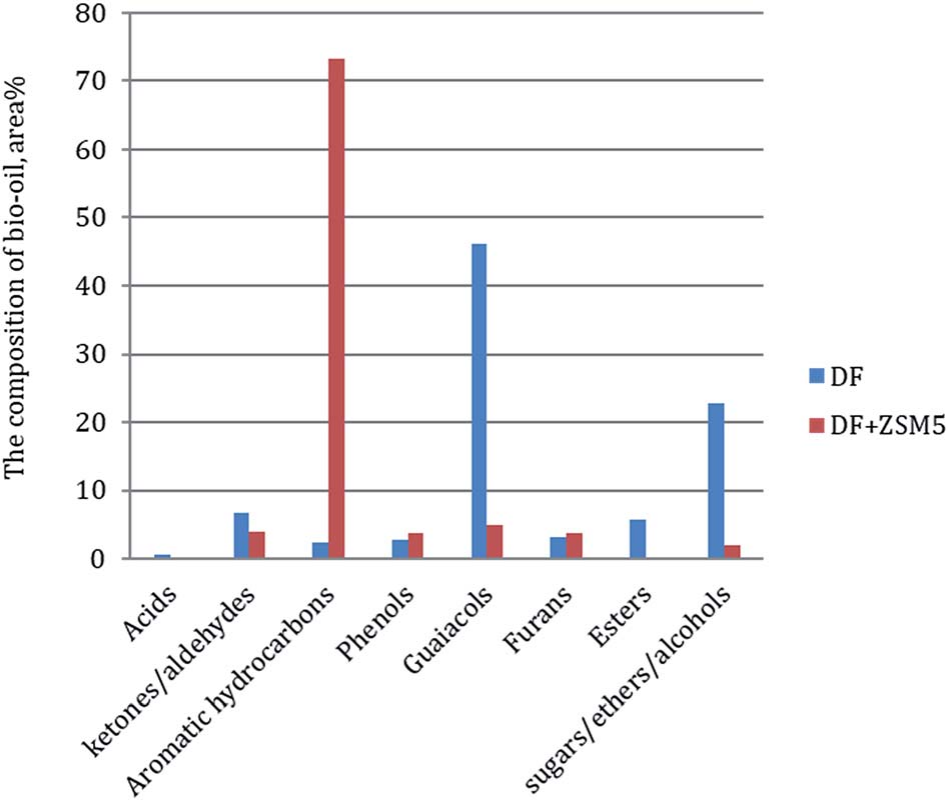
Py-GC/MS analysis of non-catalytic and catalytic pyrolysis of DF.

In order to further understand the effect of the ZSM-5 catalyst on chemical composition of bio-oil product from catalytic pyrolysis of DF, we performed Py-GC/MS to characterize the bio-oil compounds. The results were summarized to several categories based on chemical functional groups (Fig. 6). We found out that aromatic hydrocarbons, phenols and furans increased with the addition of the ZSM-5 catalyst to the pyrolysis of DF, while acids, esters, ketones/aldehydes and guaiacols exhibited the opposite tendencies. Among all the components, aromatic hydrocarbons increased significantly from 2.3 to 73.3 area% while guaiacols greatly decreased from 46.1 to 4.9 area%. The composite change of the bio-oil product showed that the catalyst had highly positive effect on aromatic hydrocarbon production by reducing oxygen-containing compounds (*i.e.* acids, esters, ketones/aldehydes and guaiacols) during DF pyrolysis. These changes of bio-oil composition were attributed to secondary cracking occurring on the surface of the ZSM-5 catalyst during the catalytic pyrolysis process.^17^ During the secondary cracking process, propenyl-guaiacols generated through the depolymerization and dehydration of DF lignin could enter the small pore-size of ZSM-5 catalyst and then converted into compounds with aromatic rings and C–H bonds (*i.e.* aromatic hydrocarbons).^37^

## 4. Conclusions

In this study, we report the thermal decomposition behavior and kinetics of pyrolysis and catalytic pyrolysis of DF through TG. According to TG and DTG curves, it was observed that the addition of the ZSM-5 catalyst tended to slightly reduce the biomass thermal degrading temperature. We found that the ZSM-5 catalyst increased the rate of reaction and reduced the energy required for decomposition process. Simulation of DF pyrolysis and catalytic pyrolysis using the obtained kinetic parameters and comparison with experimental data are in good agreement with experimental data. We further characterized the chemical composition of the bio-oil product *via* Py-GC/MS. The results showed that the ZSM-5 catalyst had a highly positive effect on aromatic hydrocarbon production by greatly reducing oxygen-containing compounds during DF pyrolysis. Therefore, in conclusion, we believe that catalytic pyrolysis of DF using ZSM-5 catalyst may be regarded as a promising method for production of high quality bio-oil.

## Conflicts of interest

There are no conflicts to declare.

## Supplementary Material
